# Effect of Ingestion of Medium-Chain Triglycerides on Substrate Oxidation during Aerobic Exercise Could Depend on Sex Difference in Middle-Aged Sedentary Persons

**DOI:** 10.3390/nu13010036

**Published:** 2020-12-24

**Authors:** Naohisa Nosaka, Shougo Tsujino, Kazumitsu Honda, Hiromi Suemitsu, Kazuhiko Kato, Kazuo Kondo

**Affiliations:** 1 Central Research Laboratory, The Nisshin OilliO Group, Ltd., Kanagawa 235-8558, Japan; s-tsujino@nisshin-oillio.com; 2 The Nisshin OilliO Group, Ltd., 1 Shinmori-Cho, Isogo-ku, Yokohama, Kanagawa 235-8558, Japan; k-honda@nisshin-oillio.com; 3 Kato Clinic, 1-1-1 Nakaizumi, Komae, Tokyo 201-0012, Japan; kato.kaz@me.com (H.S.); kato-kaz@db3.so-net.ne.jp (K.K.); 4 Ochanomizu University, 2-1-1 Ohtsuka, Bunkyo-ku, Tokyo 112-8610, Japan; k24kondo523@gmail.com; 5 Toyo University, 1-1-1 Izumino, Itakura-machi, Ora-gun, Gunma 374-0193, Japan

**Keywords:** sedentary, sex difference, aerobic exercise, exercise intensity, ventilation threshold, fat oxidation, octanoate, decanoate

## Abstract

Fat oxidation (FAO) during aerobic exercise and whole-body FAO via lipid intake are thought to be important for the maintenance of health, such as the prevention of type 2 diabetes and obesity in sedentary persons in their 40s and 50s. Medium-chain triglycerides (MCTs) ingestion has been attracting attention. However, the effects of difference of sex and the composition of medium-chain fatty acids (MCFAs) are unclear, so we examined the effects of these factors on FAO during aerobic exercise. We conducted a randomized, double-blind, placebo-controlled, 3-arm, within-participants crossover trial. FAO during low- to moderate-intensity exercise was compared when octanoate-rich MCTs (C8R), decanoate-rich MCTs (C10R), or carbohydrate (control) was ingested. Three 2-week interventions were separated by two 2-week washout periods. An increase of FAO during exercise after the C8R diet was found in males, but not in females. An increase of carbohydrate oxidation (CAO) and oxygen uptake during exercise after the C10R diet was found in females, but not in males. In a pooled estimate of the effect of MCTs (C8R and C10R) in women and men, FAO increased during exercise. In conclusion, short-term ingestion of MCTs by middle-aged sedentary persons could increase FAO during aerobic exercise compared to carbohydrate ingestion, but the enhancing effect of MCTs on substrate utilization and oxygen uptake might vary, depending on sex and the composition of MCFAs.

## 1. Introduction

Fat intake is considered to be important for health, such as prevention of type 2 diabetes and obesity. Fat intake enhances fat oxidation (FAO) during exercise and results in whole body FAO via increased mitochondrial biogenesis and enzyme activity [1,2]. A high-fat diet and ketone compounds, which are degradation products of fats, have been reported to increase FAO [3,4,5]. However, a high-fat diet causes restriction of carbohydrate intake, which has negative effects such as suppressing glycogen accumulation and increasing fatigue [6]. The use of ketone compounds has not been widespread due to their poor acceptance in taste and gastrointestinal distress [4].

Medium-chain triglycerides (MCTs) have been reported to increase FAO at relatively low doses (6 g/day) [7], and are tasteless, odorless, and as acceptable as regular fats and oils [8,9]. Animal studies have shown that the combination of aerobic exercise and MCTs ingestion increases the duration time of moderate-intensity exercise and activity of the ketone body oxidation enzyme in muscle tissue compared to the combination of aerobic exercise and long-chain triglycerides (LCT) ingestion [10]. In a human study, continuous ingestion of MCTs in recreational athletes was found to increase FAO [7] and exercise time to exhaustion [7,11].

A previous study using animals reported that MCTs ingestion without exercise also increased the duration time of moderate-intensity exercise associated with enhancement of fat utilization [10,12]. However, little is known about studies that examine the effects of FAO during aerobic exercise after ingestion of MCTs in persons without exercise habits. Furthermore, sex differences in FAO during exercise have been revealed [1,13,14], but the effects of MCTs ingestion on sex differences have not been clarified. Furthermore, a study using cultured cells examining changes in the activity of mitochondrial enzymes has reported that decanoate (C10) enhanced their activity, while octanoate (C8) did not affect it [15,16]. On the other hand, it has been reported that β-hydroxybutyrate (β-HB), which is one of the ketone bodies, increases the activity of mitochondrial enzymes and that C8 increases blood ketone bodies more than C10 after ingestion [17]. Therefore, although differences in the length of carbon chains of medium-chain fatty acids (MCFAs) that consist of MCTs may affect FAO during exercise, little is known about studies that have compared the effect of MCTs with different compositions of MCFAs.

In the present study, we examined whether there were sex differences in the effect of FAO during exercise between sedentary men and women after ingestion of MCTs. In addition, we examined the effects of ingestion of MCTs with different compositions of MCFAs.

## 2. Materials and Methods

### 2.1. Ethics

The present study was conducted in accordance with the Declaration of Helsinki (2013) and with the approval of the Ethics Committees of Nihonbashi Egawa Clinic (Ethical approval code: RD07001KW04). In advance, the study protocol was registered (UMIN000033886) with the University Hospital Medical Information Network Center (UMIN-CTR).

### 2.2. Participants

Seventeen Japanese females (Body Mass Index (BMI) 18.8–26.0) and 13 males (BMI 20.7–27.5) aged 40–59 years volunteered for the study. Participants had to have no chronic diseases, not be pregnant or lactating, and be non-smoking. Participants also had to be sedentary (less than 60 min of exercise per week and without high-labor occupational activity of 10 or more hours per week) and without contraindications for intense exercise and with a stable weight (within 10 kg for the previous 12 months). The power calculation was based on results from a previous MCT study [7]. From the result of FAO during exercise, the mean difference was 1.6 and the SD was 2.7. From crossover design power analysis, required number of participants was calculated as 13 (the following coefficient was input: alpha = 0.05, power = 0.80, theta = 1.0). An independent clinical research organization managed the participants during the intervention period and measured exercise trials. After their informed consent was obtained, screening of the participants (interview, physical measurement, blood collection and analyses, and a stepping exercise using a platform and electrocardiography) occurred as described previously [18]. To select eligible participants, the participants were screened based on the following inclusion and exclusion criteria, which were registered with UMIN-CTR (https://upload.umin.ac.jp/cgi-open-bin/ctr_e/ctr_view.cgi?recptno=R000038644 [19]).

The inclusion criteria were: (1) Japanese males and females aged 40 years or older and under 60 years at the time informed consent was obtained, and Japanese males and females who met the following inclusion criteria for protection of human rights and who did not conflict with the exclusion criteria and could comply with the management requirements during the study period; (2) subjects with a BMI of 20.7 to 27.5 in males and 18.8 to 26.0 in females (to recruit Japanese subjects with average BMI); (3) nonsmoker; (4) persons who have received sufficient explanation on the purpose and content of the research, who have the ability to give consent, who voluntarily volunteer to participate in the research with good understanding, and who have given written consent to participate in the research.

The exclusion criteria were: (1) currently receiving any medication or ambulatory treatment; (2) have a history of or complication of serious heart, liver, kidney, cardiovascular system, or blood disorders; (3) have experienced chest pain or vein abnormalities at rest; (4) frequent shortness of breath, light-headedness, dizziness, and loss of consciousness; (5) have a history of drug allergy, food allergy, or allergy to raw materials (milk protein, etc.) used in test foods; (6) have a family member who died suddenly for unknown reasons; (7) diagnosed with lumbar foot disorders; (8) taking health foods, supplements, or drugs that may affect fatigue relief; (9) eat extremely unbalanced meals; (10) extremely irregular lifestyle habits such as diet and sleep; (11) suspected of having insomnia (insomnia, sleep apnea syndrome, etc.); (12) present or past history of psychiatric disorder (depression, etc.); (13) drug dependence, present illness of alcoholism or previous history; (14) currently participating in other clinical trials or have participated in other clinical trials within the past 3 months; (15) irregular working hours, such as working at night; (16) feels an effect or pain in the lower back, knee, or body during ascent or descent of stairs, etc.; (17) receiving treatment for rheumatoid arthritis; (18) have had surgery or disease of the knee or routinely use a walking cane; (19) body weight fluctuates by ± 10 kg or more within 1 year; (20) wishing to become pregnant or are pregnant or lactating during the study period; (21) difficulties in observing records on various questionnaires; (22) exercising to maintain or improve physical fitness for at least 60 min per week; (23) engaged in physical labor for 10 h or more per week; (24) scheduled to donate blood or receive a vaccination or wishing to donate blood or receive a vaccination during the study period; (25) other, persons who are judged by medical doctor to be inappropriate for the study.

### 2.3. Randomized Allocation

The study participants were allocated by independent assignment–organization to one of three allocation arms by stratified randomization, with sex and age as a stratified factor. The results of the allocation showed that sex and age as a stratified factor were almost balanced in the three allocated groups. In three allocations, ingestion of test drink with oil was conducted in the following order (i, ii, iii); allocation 1: (i) octanoate-rich MCTs (C8R), (ii) control (carbohydrate), and (iii) decanoate-rich MCTs (C10R); allocation 2: (i) C10R, (ii) C8R, and (iii) control; allocation 3: (i) control, (ii) C10R, and (iii) C8R. The assignment information was not disclosed to the participants or the practitioner until the study was completed and the statistical analysis protocol was prepared. The study was conducted in a randomized, double-blind, and within-participants crossover manner.

### 2.4. Test Drink

The test drink provided to participants is shown in Table 1. C8R and C10R were comprised of MCTs comprising 75% of C8 and 25% of C10 (C8R) or 30% of C8 and 70% of C10 (C10R). The study participants were asked to ingest the test drink (0 g or 6 g per day of MCTs) each day. The test drinks were provided to the participants, and neither the participants nor the experiment staff were aware of the content of the drinks.

### 2.5. Intervention

**Figure 1:**
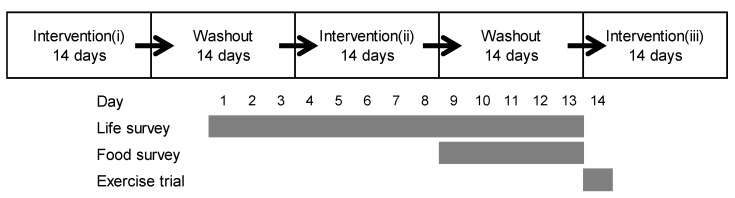
The protocol of the present study. This study was carried out in crossover manner. The subjects were examined on 3 interventions separated by a washout period of 14 days. In the intervention, the participants were asked to ingest test drink (containing 0 g of medium-chain triglycerides (MCTs) as control or 6 g of octanoic-acid rich MCTs or 6 g of decanoic-acid rich MCTs) and to record their consumption each day for 13 days. They were instructed to maintain their physical activity at a fixed level and to record their activity time for 13 days. They were also asked to record all meals consumed from days 9 to 13 and to abstain from exercise and alcohol on day 13. On day 14, exercise trial was conducted in each intervention. Intervention (i); the first period of intervention; intervention (ii): the second period of intervention; intervention (iii): the third period of intervention.

This study included three 14-day interventions separated by two 14-day washout periods (Figure 1). The details of the interventions were described previously [18]. In brief, the 14-day interventions consisted of a 13-day life survey, 5-day food survey, and 1-day exercise trial. Nutritional calculations were performed by the managing registered dietitian to obtain daily intakes of energy, protein, lipid, carbohydrate, and MCFAs (C8 and C10) from the dietary records (photographs, with a ruler and food survey sheet) on the basis of the standard tables of food composition in Japan, 2015 (seventh revised edition) [20].

### 2.6. Exercise Trial

On day 14 the participants were asked to visit the laboratory and underwent an exercise trial. The details of the procedure of the exercise trial were previously shown [18]. In brief, the participants were asked to measure oxygen uptake (VO2) and carbon dioxide excretion (VCO2) in a sitting posture for five minutes before the experimental trial (rest), using a respiratory gas analyzer (AE-310S; Minato Medical Science, Osaka, Japan). The participants wore the mask used for the expired gas measurement in such a way that no expired air leaked from the mask. The participants were first required to exercise on a stationary bicycle at a pedaling frequency (cadence) of 50−60 rpm and at a fixed workload of 20 watts (20-watt fixed-load exercise (20Ex)) for 3 min. The workloads were then incrementally increased by 13 watts (male) and 10 watts (female) per min (incremental load exercise (InEx)) until their ventilation threshold (VT) was reached. The appearance of VT was defined as the point when the respiratory exchange ratio (RER) rapidly exceeded 1.00 by the V-slope method [21].

### 2.7. Calculation

From respiratory measurements (VO2 and VCO2), RER, FAO, and carbohydrate oxidation (CAO) rates were calculated as follows [22,23].

RER = VCO2/VO2(1)

FAO rate = 1.695 VO2 − 1.701 VCO2(2)

CAO rate = 4.585 VCO2 − 3.226 VO2(3)

The cumulative value of FAO and CAO (cFAO and cCAO) was calculated from the FAO and CAO rates during 20Ex, for 2 min (from 2 to 3 min after starting the exercise), and during InEx until the appearance of VT. The maximal FAO rate (mFAO) was determined during 20Ex, for 2 min (from 2 to 3 min after starting), and during InEx. The RER at mFAO was determined during 20Ex (RER at mFAO during 20Ex (RER@20Ex), (@, at time of)) and during InEx (RER at mFAO during InEx (RER@InEx)).

Power outputs at VT (PO@VT) during InEx were determined. VO2 at VT (VO2@VT) during InEx were determined. Ventilation volume per VCO2 (VE/VCO2) at VT (VE/VCO2@VT) during InEx were calculated as the ventilation volume (unit: ml/min) divided into the VCO2 volume (unit: mL/min). Values of FAO, power outputs (PO), and RER were determined as moving averages (every 1 min) calculated from the expired gas measurement value taken every 10 s. The average and variance of cadence (unit: rpm) were calculated every minute, from 20Ex to InEx. To estimate the exercise intensity of the present study, the ratio of energy expenditure before (while resting) and during exercise was calculated. Abbreviations are described in the Abbreviations Section.

### 2.8. Statistical Analyses

A statistical analysis protocol was prepared before the disclosure of allocation information. After obtaining their informed consent, qualified participants were determined by screening test. The qualified participants who did not withdraw their consent before the start of the intervention became the study participants, and the data were made into a full analysis set (FAS).

Of the FAS, participants disqualified by analysis were determined using the following criteria: (i) those whose consent was withdrawn after starting the intervention; (ii) those whose consumption of the test food did not meet the following criteria: in each intervention, those who had taken fewer than two packages of the test food for two consecutive days, or those who had not consumed fewer than two packages of the test food for three days; (iii) those who had not sufficiently provided the records requested during the intervention; (iv) in the exercise trial, those who did not attend for personal reasons; (v) those who stopped pedaling or whose cadence dropped below 50 rpm three times during the exercise trial; and (vi) those who discontinued pedaling before the appearance of VT.

In the event of any participants disqualified by analysis, the data of analysis-qualified participants would be made per protocol set (PPS). The study participants were divided into two sex groups, and the following statistical analysis was conducted on FAS and PPS.

The bodyweight data in the intervention and for the data obtained from the exercise trial (cadence, cFAO, cCAO, mFAO, RER@20Ex, RER@InEx, PO@VT, VO2@VT, and VE/VCO2@VT) were calculated as the difference, (C8R diet or C10R diet) minus (control diet), as intervention effect. The intervention effects were confirmed by the Shapiro–Wilk test for normality of distribution. When the normality of the data was confirmed, the data were compared using a linear mixed model for repeated measures in which allocation and intervention period were set as fixed effect and participants were set as a random effect, and when normality was not confirmed, the Bartlett’s test was used to confirm equal variance. When equal variance was confirmed, then Dunnett’s method was performed. When equal variance was not confirmed, Steel’s test was performed.

Regarding the mean values of nutritional daily intake during intervention (energy, protein, fat, carbohydrate, and MCFAs (C8 and C10)), multiple comparisons were performed to compare the control group with the test groups. When equal variance was confirmed by Bartlett’s test, then Dunnett’s method was performed. When equal variance was not confirmed, Steel’s test was performed.

All statistical measurement values were expressed as mean and standard deviation or standard error or 95% confidence intervals. Calculation of basic statistics of the data was performed using Microsoft Excel 2010 for Windows (Microsoft Japan Co., Ltd., Tokyo, Japan). All statistical analyses were performed using R statistical software, v3.4.3 for Windows (R Core Team, Vienna, Austria) [24]. The level of significance for all comparisons was set at *p* < 0.05.

An exploratory group analysis was conducted to estimate the overall pooled effect and its confidence of cFAO. The pre-specified group was sex and diet. The mean difference and 95% confidence interval of cFAO in intervention effect during overall (20Ex and InEx; VT) exercise for each group, and the pooled estimate of cFAO, were calculated by a random effects model with a restricted maximum likelihood method.

## 3. Results

Part of the data (unstratified data and age-stratified data) obtained in the present study has been published previously [18], and analyses of the sex-stratified data on sex difference were conducted in the present study.

### 3.1. Participants

**Figure 2:**
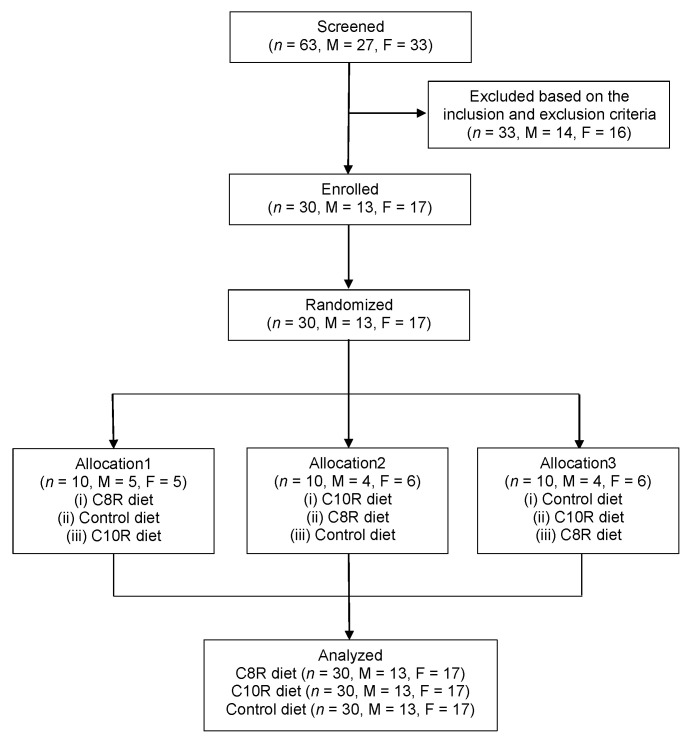
Flowchart illustrating the selection and participation of study participants in the present study. Thirty-six female and 27 male participants underwent screening, which included a medical history, physical, and blood tests. Of the 63 participants, 19 female and 14 male participants were not assigned. The remaining 17 female and 13 male participants were randomly allocated to 3 groups to receive the intervention (control diet: 0 g of MCTs; C8R diet: 6 g of octanoic-acid rich MCTs; C10R diet: 6 g of decanoic-acid rich MCTs). No participants dropped out and were excluded. The data from 17 female and 13 male participants were analyzed. F: females; M: males; C8R: octanoate-rich MCTs; C10R: decanoate-rich MCTs.

Figure 2 is a flowchart of study participants. Of the 13 male and 17 female eligible participants who were enrolled, no participants dropped out before or after randomization. All study participants completed the study, and there were no analysis-disqualified participants. Hence, the data were analyzed using the FAS. The characteristics of the present study participants are shown in Table 2.

### 3.2. Dietary Intake

Mean average dietary intakes per day for the five days before each exercise trial are shown in Table 3. In both males and females, there were no significant differences in energy, protein, carbohydrate, and fat intakes in the C8R and C10R diet, compared to the control diet. MCFAs (C8 and C10) intakes were significantly greater in the C8R and C10R diet than in the control diet.

### 3.3. Exercise Trial

The means and variances of body weights during the intervention, and of cadence during the exercise trials, were compared between the diet groups. No significant difference was found. The ratios of 20Ex and VT to rest were 2.2 ± 0.1 times and 4.5 ± 0.9 times (mean ± SD) in female participants.

The intervention effect of ingestion of MCFAs, which expresses values (C8R or C10R diet) minus (control diet), is shown in Table 4. The normality distribution on the data of intervention effect is shown in Table A1. For female participants ingesting the C8R and C10R diet, there were no changes to cFAO, mFAO, and RER during 20Ex, InEx, and overall (20Ex and InEx until VT); there were changes to cCAO during InEx and overall, but not 20Ex. When ingesting the C10R diet, PO@VT, VO2@VT, and overall elapsed time were all greater, but not when consuming the C8R diet (Table 4).

For male participants ingesting the C8R diet, cFAO during 20Ex and PO@VT were significantly greater, and cCAO during 20Ex, RER@20Ex, and RER@InEx were significantly less; there were no changes to PO, elapsed time, VO2, and VE/VCO2; there were no changes when ingesting C10R (Table 4).

For female and male participants, the intervention period effects of the measures that were significant for the allocation (diet group) were not significant.

**Figure 3:**
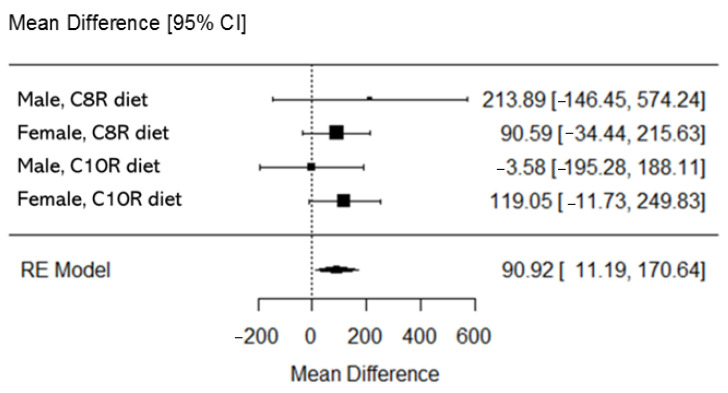
Pooled analysis of cumulative value of fat oxidation during overall (20Ex and InEx, ~VT) exercise in the present study. Values are mean difference and 95% coefficient interval. Random effect model with restricted maximum likelihood method was performed.

In the pooled analysis of cFAO in intervention effect during overall exercise (20Ex and InEx, ~VT), the mean difference and 95% confidence interval (CI) for the pooled estimate were 90.92 mg (11.19 to 170.64 mg, 95% CI, *p* < 0.05) (Figure 3). I^2^ (degree of heterogeneity) was 0.00%, and the Q test (degree of freedom = 3) was 1.5587 (*p* = 0.669) for the test of heterogeneity. In the funnel plot analysis, z was 0.178 (*p* = 0.859) in the test for funnel plot asymmetry. Kendall’s tau was 0.333 (*p* = 0.750) for the rank correlation test for funnel plot asymmetry.

## 4. Discussion

MCTs have been used to enhance FAO during exercise because they are rapidly metabolized after ingestion and are hardly stored [8,25]. Previous studies in which MCTs were ingested before exercise showed negative results [10,26,27]. In a study in which MCTs enhanced fat utilization during exercise, it was effective up to one hour after ingestion, but not two hours [28]. Studies that combined pre-exercise MCTs ingestion and intermittent MCTs ingestion during exercise increased FAO during exercise [22,29] and improved performance during moderate-intensity exercise followed by high-intensity exercise [29]. However, in several studies FAO and performance did not improve during high-intensity exercise [23,30] and resulted in gastrointestinal distress [23]. A study investigating FAO during exercise with intravenous octanoate at moderate and high intensities showed that oxidation of MCFAs in peripheral blood was not inhibited regardless of intensity [31], suggesting that FAO ability during exercise affected the oxidation of MCFAs. It is well known that training improves FAO capacity [32], and it is believed that MCTs ingestion in trained people with high FAO capacity may be more useful than LCT ingestion in supplying fat fuel. However, MCTs intake before and during exercise in people who are without exercise habits with low FAO capacity is unlikely to be useful. Therefore, MCTs ingestion methods that enhance FAO capacity are crucial for people without exercise habits. Short-term continuous ingestion of MCTs did not enhance FAO in high-intensity exercise [33,34] but did enhance FAO in low- to moderate-intensity exercise [7,18]. It has also been reported that gastrointestinal discomfort symptoms should be noted when MCTs intake exceeds 17 g at a time [33], but 6 g intake has little effect in middle-aged sedentary persons [18]. The present study aimed to evaluate people without exercise habits on substrate oxidation during exercise with a fixed load of 20W after continuous MCTs ingestion. The 20-watt exercise was approximately 2.2 times the metabolic rate when at rest and was lower than 3.0–5.9 times, which is the definition of moderate-intensity exercise [35], so it was considered to correspond to low-intensity exercise. Furthermore, the incremental-load exercise until VT (to resting, approximately 4.5 times at VT) was used because of the low aerobic-exercise capacity in people without exercise habits and the variability in the appropriate exercise load for each individual in aerobic exercise [1].

An exploratory pooled analysis to estimate the influences of all MCTs (C8R and C10R) and sex (female and male) resulted in a positive effect of cFAO during low- to moderate-intensity aerobic exercise in middle-aged sedentary persons who do not exercise habitually. Previous study results [7,18] and the result of the present study support the proposal that enhancement of FAO during aerobic exercise by short-term MCTs ingestion could not depend on exercise habit. However, the results of the sex-specific evaluation showed that there was a sex difference in the increase of FAO and CAO during aerobic exercise after continuous ingestion of MCTs between women and men, as women were presumed to promote both FAO and CAO via increased oxygen uptake, while men were presumed to inhibit CAO instead of promoting FAO. When women consumed MCTs, they showed a trend toward increased FAO during InEx (*p* = 0.06, for the C10R diet) without affecting FAO during 20Ex. This result indicates that the effect of MCTs ingestion may not have been observed in women due to their higher daily FAO, which may be related to the higher daily fat utilization in women than in men, as pointed out in previous studies [1,13]. Furthermore, the results of the evaluation using incremental exercise until VT showed an increase in oxygen uptake in women, and this increase in oxygen uptake was associated with a significant increase in carbohydrate oxidation. A previous human study has reported an increase in blood triglycerides and an increase in RER during exercise after the 2-week MCTs ingestion [34], indicating that MCTs intake may promote both carbohydrate assimilation and oxidation. Furthermore, studies using animals have reported inhibition of the increase in pyruvate dehydrogenase kinase 4 protein, which suppressed the glycolytic system on a ketogenic MCTs diet [36] and increased blood glucose uptake on a high-fat MCTs diet [37]. The increase in carbohydrate oxidation obtained in the women in the present study may have been due not only to the increase in oxygen uptake by continuous MCTs ingestion but also to the enhancement of glucose metabolism shown in previous studies. When men consumed MCTs, an increase in FAO and mFAO and a decrease in RER were observed during 20Ex, and a decrease in RER was observed during InEx, indicating a possible increase in FAO during aerobic exercise. We also observed a decrease in CAO during 20Ex, but no change in oxygen uptake and CAO during InEx. The lower daily FAO in men as compared to women may be related to substrate oxidation during exercise [1].

In the present study, we examined the effects of MCTs consisting of different compositions of MCFAs on FAO during exercise. Our results indicate that the ingestion of both MCTs may increase FAO in women. On the other hand, in males, C8R ingestion clearly had a greater effect, while C10R intake might not have an effect. Since the C8/C10 ratio of commonly used MCTs (60/40) [8,9] is close to that of C8R (75/25) in this study, the increase in FAO by C8R intake observed in males might be no different from previous studies examining the effects of MCTs ingestion. In addition, regarding the effect on carbohydrate oxidation, C8R ingestion was suppressed in men, and C10R did not have an effect, but both MCTs ingestions increased in women. These results indicate that C8R ingestion may be more potent in increasing FAO during exercise than C10R ingestion and that C10R ingestion may increase CAO via increased oxygen uptake during exercise. Therefore, it was shown that substrate oxidation during exercise might be differentially affected via differences in daily substrate oxidation between men and women [1] and the composition of MCFAs in MCTs.

There are several limitations to the present study. First, the lack of a prior power analysis may have resulted in a sample size that was less than what would have enabled us to draw clear and valid conclusions in the study outcomes. In addition, the study was conducted on the average Japanese BMI level (BMI 22–24), and it is unclear whether the effect would be similar for the average BMI level in other countries. Finally, in the present study, the measurement of substrate oxidation is affected by the exercise modality, as the exercise trials were performed on a bicycle ergometer with a combination of fixed and incremental loads, and it is not clear whether our results can be translated to other exercise modalities such as running and swimming.

Before starting exercise in a person who does not habitually exercise, a self-check questionnaire and medical check are considered to be important to maintain health [38]. In addition, food-dependent exercise-induced anaphylaxis (FEIAn) is known to be an exercise-induced allergy [39]. In general, FEIAn is induced by high-intensity exercise after ingestion of foods that are wheat products or crustaceans [40].

## 5. Conclusions

An interventional study showed that short-term ingestion of MCTs by sedentary participants aged in their 40s and 50s could increase FAO during aerobic exercise compared to carbohydrate ingestion, but the enhancing effect of MCTs on substrate utilization and oxygen uptake might vary, depending on sex and the composition of MCFAs.

## Figures and Tables

**Table 1:** Test drinks in the present study.

Nutrition	Control	C8R	C10R
Energy, kcal	200	200	200
Protein, g	1.4	1.4	1.4
Fat, g	0	6.0	6.0
(C8R MCTs, g)	(-)	(6.0)	(-)
(C10R MCTs, g)	(-)	(-)	(6.0)
Carbohydrate, g	48.0	34.6	34.6

C8R: octanoate-rich MCTs; C10R: decanoate-rich MCTs; medium-chain triglycerides (MCTs); (-): not contained.

**Table 2:** Characteristics of the participants in the present study.

Characteristics	Sex	Value
Number, *n*	Female	17
	Male	13
Age, year	Female	48.2 ± 3.7
	Male	48.8 ± 3.6
Height, cm	Female	157.8 ± 5.0
	Male	169.1 ± 8.8
Body weight, kg	Female	55.4 ± 5.6
	Male	67.8 ± 9.0
Body mass index, kg/m^2^	Female	22.2 ± 1.7
	Male	23.6 ± 1.1

Values are means ± standard deviations.

**Table 3:** Means of dietary intake per day, during the five days before each exercise trial in the present study.

Nutritional Indices	Control Diet	C8R Diet	C10R Diet
Female, *n* = 17			
Energy, kcal	1758 ± 337	1755 ± 252	1775 ± 349
Protein, g	56.6 ± 13.0	57.9 ± 9.7	58.3 ± 10.6
Fat, g	58.9 ± 17.3	63.6 ± 12.6	67.9 ± 14.8
Octanoic acid, mg	124.8 ± 118.5	3790.8 ± 78.8 *	1623.4 ± 67.1 *
Decanoic acid, mg	211.5 ± 147.6	1506.2 ± 142.4 *	3724.0 ± 133.2 *
Carbohydrate, g	240.5 ± 47.6	225.5 ± 35.9	223.5 ± 52.9
Male, *n* = 13			
Energy, kcal	1978 ± 342	2034 ± 412	1936 ± 430
Protein, g	69.6 ± 19.2	70.9 ± 17.2	64.8 ± 12.4
Fat, g	63.2 ± 16.5	69.8 ± 20.5	67.0 ± 22.0
Octanoic acid, mg	88.5 ± 116.4	3748.8 ± 101.7 *	1585.4 ± 84.0 *
Decanoic acid, mg	167.3 ± 227.8	1402.6 ± 121.2 *	3657.9 ± 150.5 *
Carbohydrate, g	268.5 ± 49.9	259.7 ± 51.8	253.2 ± 55.8

Values are means ± SD. * Statistically significant difference from the value in the control diet (*p* < 0.05). If equality of the 3 variances was hypothesized when performing Bartlett’s test, Dunnett’s test was performed. If the hypothesis was rejected, Steel’s test was performed.

**Table 4:** Cumulative values of fat and carbohydrate oxidation, maximal fat oxidation rate, respiratory exchange ratio, power output, oxygen uptake, and ventilation volume per VCO2 volume during the experimental trial after ingestion of a control or C8R or C10R diet for two weeks, in the present study.

Intervention Effect, ∆	Diet	Female, *n* = 17	*p*	Male, *n* = 13	*p*
Cumulative fat oxidation, mg					
^#^ 20Ex	C8R	12.1 ± 16.2	0.46	84.0 ± 28.2	0.007
	C10R	6.5 ± 16.2	0.69	37.1 ± 28.2	0.20
^#^ InEx (~VT)	C8R	82.6 ± 55.0	0.14	118 ± 139	0.41
	C10R	108 ± 55.0	0.06	−28.6 ± 139	0.84
^#^ overall (20Ex and InEx, ~VT)	C8R	94.7 ± 65.8	0.16	202 ± 151	0.20
	C10R	115 ± 65.8	0.09	8.5 ± 151	0.96
Cumulative carbohydrate oxidation, mg					
^#^ 20Ex	C8R	9.1 ± 38.8	0.82	−286 ± 81.6	0.002
	C10R	−9.8 ± 38.8	0.80	−46 ± 81.6	0.58
^#^ InEx (~VT)	C8R	775 ± 373	0.046	−188 ± 770	0.81
	C10R	825 ± 373	0.03	366 ± 770	0.64
^#^ overall (20Ex and InEx, ~VT)	C8R	784 ± 381	0.048	−474 ± 803	0.56
	C10R	815 ± 381	0.04	320 ± 803	0.69
Maximal fat-oxidation rate, mg/min					
^#^ 20Ex	C8R	11.6 ± 8.7	0.19	38.2 ± 17.4	0.04
	C10R	1.8 ± 8.7	0.84	22.0 ± 17.4	0.22
^#^ InEx	C8R	16.6 ± 9.3	0.08	22.4 ± 21.2	0.30
	C10R	12.3 ± 8.1	0.20	9.2 ± 21.2	0.67
Respiratory exchange ratio					
^#^ 20Ex (at mFAO)	C8R	−0.009 ± 0.008	0.28	−0.05 ± 0.02	0.007
	C10R	−0.004 ± 0.008	0.63	−0.02 ± 0.02	0.30
^#^ InEx (at mFAO)	C8R	−0.007 ± 0.008	0.38	−0.03 ± 0.01	0.02
	C10R	−0.006 ± 0.008	0.48	−0.01 ± 0.01	0.27
Power output, W					
^#^ InEx (at VT)	C8R	2.5 ± 2.0	0.23	4.5 ± 4.1	0.28
	C10R	5.8 ± 2.0	0.008	4.3 ± 4.1	0.30
Elapsed time, sec					
overall (20Ex and InEx, ~VT)	C8R	15.2 ± 12.3	0.22	22.0 ± 19.1	0.26
	C10R	34.8 ± 12.3	0.008	21.1 ± 19.1	0.28
Oxygen uptake, ml/min					
^#^ InEx (at VT)	C8R	28.8 ± 31.2	0.36	−31.2 ± 78.6	0.70
	C10R	60.5 ± 27.2	0.009	67.9 ± 78.6	0.40
Ventilation volume per VCO2 volume, mL/mL					
^#^ InEx (at VT)	C8R	0.01 ± 0.43	0.99	0.56 ± 0.42	0.20
	C10R	−0.39 ± 0.43	0.37	0.77 ± 0.42	0.08

Values are expressed as intervention effect, ∆ value ((C8R or C10R diet) minus (Control diet)). Values are least squares means ± SE. 20Ex: 20-watt fixed-load exercise; InEx: incremental load exercise; (^#^) overall: from 20Ex to InEx until VT; VT: ventilation threshold; mFAO: maximal fat oxidation.

## Data Availability

Data not available due to commercial restrictions.

## Abbreviations

The abbreviation list of measurement in exercise trial.

FAO	Fat oxidation
CAO	Carbohydrate oxidation
RER	Respiratory exchange ratio
VT	Ventilation threshold
cFAO	Cumulative value of FAO
cCAO	Cumulative value of CAO
mFAO	Maximal FAO rate
PO	Power output
VE/VCO2	Ventilation volume per VCO2
@	At time of

## Appendix A

**Table A1:** Statistical analyses of normality distribution on the data of intervention effect.

Intervention Effect, ∆	Diet	Female, *n* = 17W	*p*	Male, *n* = 13W	*p*
Cumulative fat oxidation, mg					
20Ex	C8R	0.98	0.95	0.95	0.55
	C10R	0.97	0.74	0.93	0.37
InEx (~VT)	C8R	0.96	0.57	0.94	0.46
	C10R	0.95	0.39	0.83	0.02
^#^ overall (20Ex and InEx, ~VT)	C8R	0.95	0.53	0.96	0.73
	C10R	0.92	0.14	0.93	0.31
Cumulative carbohydrate oxidation, mg					
20Ex	C8R	0.93	0.21	0.96	0.69
	C10R	0.96	0.67	0.93	0.33
InEx (~VT)	C8R	0.97	0.85	0.96	0.72
	C10R	0.93	0.18	0.91	0.18
^#^ overall (20Ex and InEx, ~VT)	C8R	0.97	0.89	0.97	0.86
	C10R	0.90	0.06	0.92	0.22
Maximal fat-oxidation rate, mg/min					
20Ex	C8R	0.96	0.65	0.94	0.51
	C10R	0.98	0.98	0.92	0.22
InEx	C8R	0.95	0.53	0.94	0.51
	C10R	0.88	0.03	0.93	0.36
Respiratory exchange ratio					
20Ex (at mFAO)	C8R	0.97	0.89	0.97	0.85
	C10R	0.97	0.78	0.93	0.30
^#^ InEx (at mFAO)	C8R	0.99	0.99	0.97	0.93
	C10R	0.96	0.67	0.97	0.92
Power output, W					
InEx (at VT)	C8R	0.97	0.82	0.93	0.29
	C10R	0.93	0.21	0.96	0.81
Elapsed time, sec					
overall (20Ex and InEx, ~VT)	C8R	0.97	0.83	0.93	0.39
	C10R	0.92	0.17	0.97	0.83
Oxygen uptake, ml/min					
InEx (at VT)	C8R	0.96	0.57	0.87	0.05001
	C10R	0.87	0.02	0.96	0.73
Ventilation volume per VCO2 volume, mL/mL					
InEx (at VT)	C8R	0.94	0.36	0.96	0.79
	C10R	0.92	0.16	0.99	1.00

20Ex: 20-watt fixed-load exercise; InEx: incremental load exercise; (^#^) overall: from 20Ex to InEx until VT; VT: ventilation threshold; mFAO: maximal fat oxidation.
